# Supplementary material for: A Serine Protease Inhibitor, Camostat Mesilate, Suppresses Urinary Plasmin Activity and Alleviates Hypertension and Podocyte Injury in Dahl Salt-Sensitive Rats
Source: Int J Mol Sci. 2023 Oct 30;24(21):15743. doi: 10.3390/ijms242115743 (PMC10650472; doi:10.3390/ijms242115743)
Supplement: Supplementary file 1 [file ijms-24-15743-s001.zip › ijms-2676397-supplementary.pdf]

Supplementary Figure S1

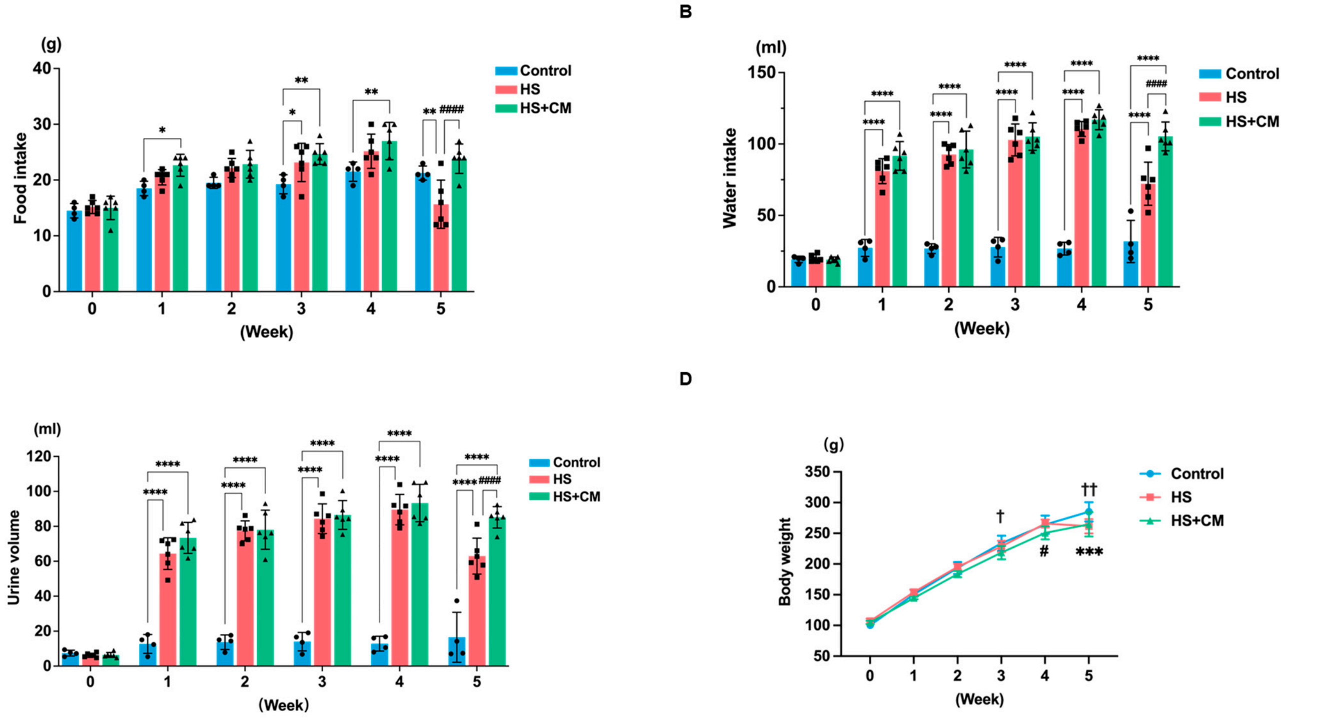

Figure S1. General parameters in the Control, high-salt (HS), and HS+camostat mesilate (CM) groups. (A) Food intake, (B) water intake, (C) urine volume, and (D) body weight (BW) measured weekly; n = 4, 6, and 6 for the Control, HS, and HS+CM groups, respectively. The results were reported as the average and the standard deviation. We analyzed results using two-way ANOVA, followed by the Tukey test. *: P < 0.05, **: P < 0.01, ***: P < 0.001, ****P < 0.0001 vs Control group. #: P < 0.05, ##: P < 0.01 vs HS group. †: P < 0.05, ††: P < 0.01, Control group vs HS+CM group.

Supplementary Figure S2

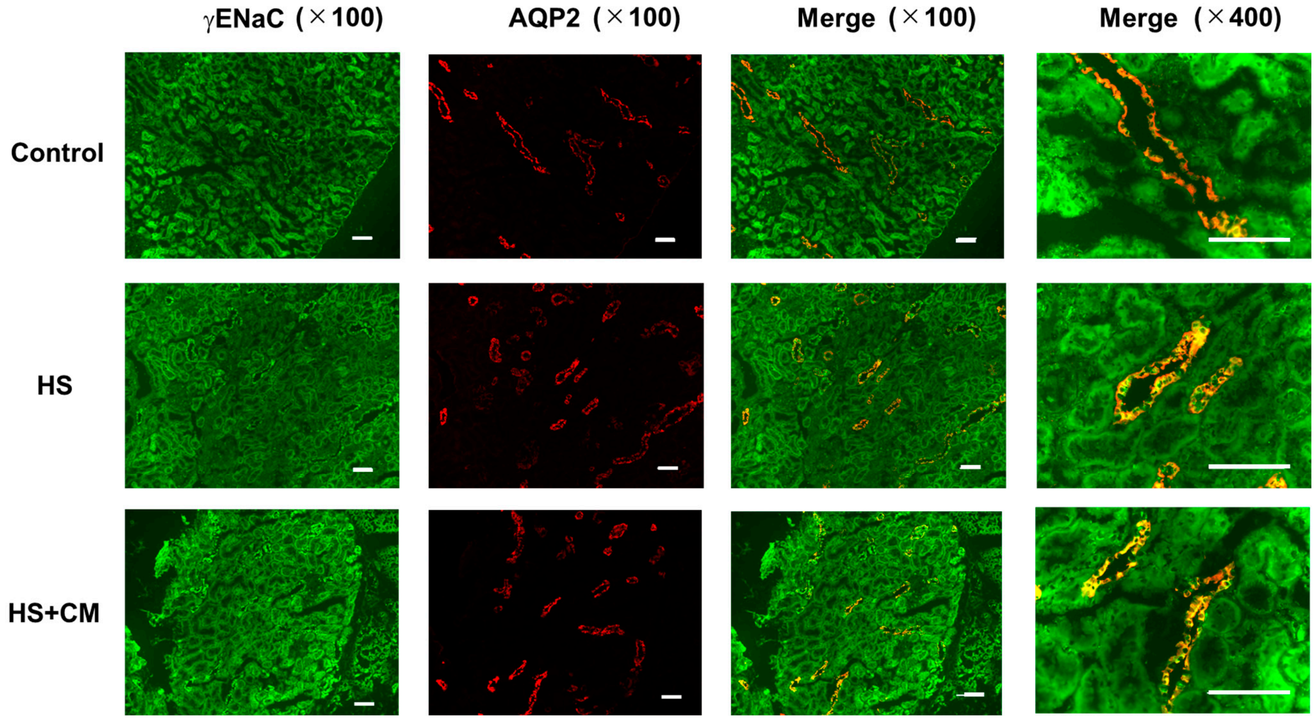

Figure S2. Double immunofluorescence staining of γENaC and AQP2.

The immunofluorescent staining confirmed the co-localization of γENaC and AQP2 in the collecting duct cells. The HS loading induced the apical trafficking of γENaC, which was not apparently affected by CM treatment. White scale bar represents 100 μm.

Supplementary Figure S3

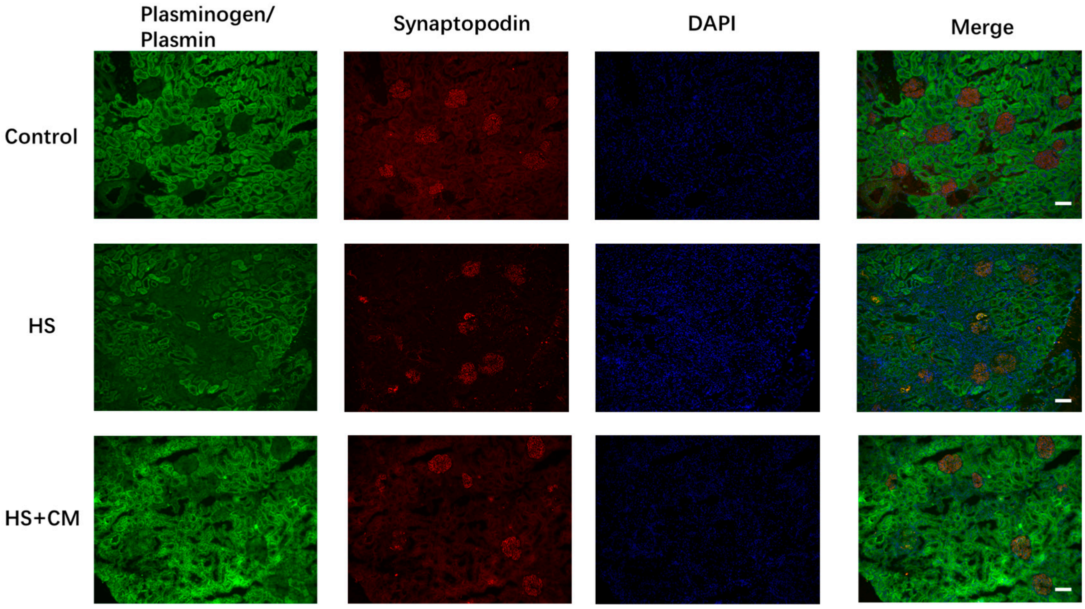

Figure S3. Lower magnification (100× magnification) of immunofluorescence staining of plasminogen/plasmin and synaptopodin.

In the HS group, plasminogen/plasmin adheres to the glomerulus and co-localizes with synaptopodin, which appears reduced due to glomerular injury. These changes are mitigated by CM treatment. White scale bar represents 100 μm.
